# Supplementary material for: From sequence to enzyme mechanism using multi-label machine learning
Source: BMC Bioinformatics. 2014 May 19;15:150. doi: 10.1186/1471-2105-15-150 (PMC4229970; doi:10.1186/1471-2105-15-150)
Supplement: Additional file 2 — Java code of ml2db. Additional file ml2db_code.tar.gz contains the Java source code to run the multi-label machine learning experiments and save the results to database. The code’s Javadoc is included. [file 1471-2105-15-150-S2.zip › additional file 2/ml2db/ml2db/doc/main/ml2db/al/utility/confidence/package-use.html]

Uses of Package main.ml2db.al.utility.confidence


JavaScript is disabled on your browser.


- Overview
- Package
- Class
- Use
- Tree
- Deprecated
- Index
- Help

- Prev
- Next

- Frames
- No Frames

- All Classes

# Uses of Package main.ml2db.al.utility.confidence

- Packages that use main.ml2db.al.utility.confidence

  | Package | Description |
  | --- | --- |
  |  |  |
  | --- | --- |
  | main.ml2db.al.utility.confidence |  |
  | main.ml2db.al.utility.hybrid |  |
  | test.ml2db.al.utility.confidence |  |
- Classes in main.ml2db.al.utility.confidence used by main.ml2db.al.utility.confidence

  Class and Description

  ConfidenceCalculator

  Gets the confidence of a multi-label prediction and calculates how close it
  is to 50% (maximum uncertainty)

  ConfidencesStorage
- Classes in main.ml2db.al.utility.confidence used by main.ml2db.al.utility.hybrid

  Class and Description

  ConfidenceCalculator

  Gets the confidence of a multi-label prediction and calculates how close it
  is to 50% (maximum uncertainty)
- Classes in main.ml2db.al.utility.confidence used by test.ml2db.al.utility.confidence

  Class and Description

  ConfidenceCalculator

  Gets the confidence of a multi-label prediction and calculates how close it
  is to 50% (maximum uncertainty)

  ConfidenceMetric

  Combines a Positioning metric (a metric that considers positively if an
  instance is close to other unlabelled instances and distant from other
  labelled instances) with the average certainty of the instance prediction
  (that is, how distant the average of confidence over all labels is from 50%)

- Overview
- Package
- Class
- Use
- Tree
- Deprecated
- Index
- Help

- Prev
- Next

- Frames
- No Frames

- All Classes
